# Supplementary material for: Ligand-induced type II interleukin-4 receptor dimers are sustained by rapid re-association within plasma membrane microcompartments
Source: Nat Commun. 2017 Jul 14;8:15976. doi: 10.1038/ncomms15976 (PMC5519985; doi:10.1038/ncomms15976)
Supplement: Supplementary Information — Supplementary Figures, Supplementary Tables, Supplementary Methods, Supplementary References. [file ncomms15976-s1.pdf]

Type of file: pdf
Size of file: 0 KB
Title of file for HTML: Peer Review File
Description:

Type of file: pdf
Size of file: 0 KB
Title of file for HTML: Supplementary Information
Description: Supplementary Figures, Supplementary Tables, Supplementary Methods, Supplementary References.

Type of file: avi
Size of file: 0 KB
Title of file for HTML: Supplementary Movie 1
Description: Co-tracking of ^{DY547}IL-4Rα-TM and ^{DY647}IL-13Rα1-TM co-reconstituted in PSM and incubated with different concentrations of KFR (concentrations indicated in each panel). Cotrajectories are colored in magenta.

Type of file: mp4
Size of file: 0 KB
Title of file for HTML: Supplementary Movie 2
Description: TIRF microscopy imaging of individual ^{DY647}KFR bound to endogenous IL-4Rα in HeLa cells (imaged at 31.25 Hz, playback speed: real time). Left: overview of an entire cell. The boundary of the cell is outlined by a white line. Right: zoom-up including trajectories on individual ^{DY647}KFR in red.

Type of file: mp4
Size of file: 0 KB
Title of file for HTML: Supplementary Movie 3
Description: Co-locomotion of an individual IL-4Rα/IL-13Rα1 signaling complex measured in presence of IL-4 under steady-state conditions (imaged at 31.25 Hz, playback speed: real time, colocalization threshold: 214 nm). Left: overlay of trajectories (^{DY647}IL-4Rα: green, ^{TMR}IL-13Rα1: magenta, cotrajectory: white, scale bar = 1 µm). Right: Distance between both receptor subunits within this trajectories.

Type of file: mp4
Size of file: 0 KB
Title of file for HTML: Supplementary Movie 4
Description: Dynamics (dissociation and re-association events) of an individual IL-4Rα/IL- 13Rα1 signaling complex measured in presence of RGA under steady-state conditions (imaged at 31.25 Hz, playback speed: 0.33× real time, co-localization threshold: 214 nm). Left: overlay of trajectories (^{DY647}IL-4Rα: green, ^{TMR}IL-13Rα1: magenta, co-trajectory: white, scale bar: 1 µm). Right: Distance between the receptor subunits within these trajectories.

Type of file: mp4
Size of file: 0 KB
Title of file for HTML: Supplementary Movie 5

Description: Diffusion And Interaction Dynamics Simulation (DAIDS). Representative simulation run with parameter-set A and a dissociation probability corresponding to wt k_d^T, limited to a simulation length of 30 s. Image creation and bleaching of particles starts after 300 frames (9.6 s). For a more realistic PICCS live evaluation during a simulation run, particle positions are blurred according to a localization precision of 25 nm before transferring to the PICCS algorithm. Axis labeling of the simulated surface in pixel (0.107 µm per pixel). Labeled receptor subunits are depicted as filled dots, unlabeled and bleached receptor subunits as hollow dots (green: ^{DY647}IL-4Rα; magenta: ^{TMR}IL-13Rα1). Ternary complexes with intact labels on both receptor subunits are indicated by a magenta dot surrounded by a white halo. Ternary complexes with one or both labels bleached are indicated by a filled dot surrounded by a hollow dot.

Type of file: mp4
Size of file: 0 KB
Title of file for HTML: Supplementary Movie 6
Description: Experimental vs. simulated receptor movement. Left: TIRF microscopy imaging of individual ^{DY647}KFR bound to endogenous IL-4Rα in the plasma membrane of a HeLa cell. Right: Image stack created with DAIDS. Imaged at 31.25 Hz, playback speed: real time.

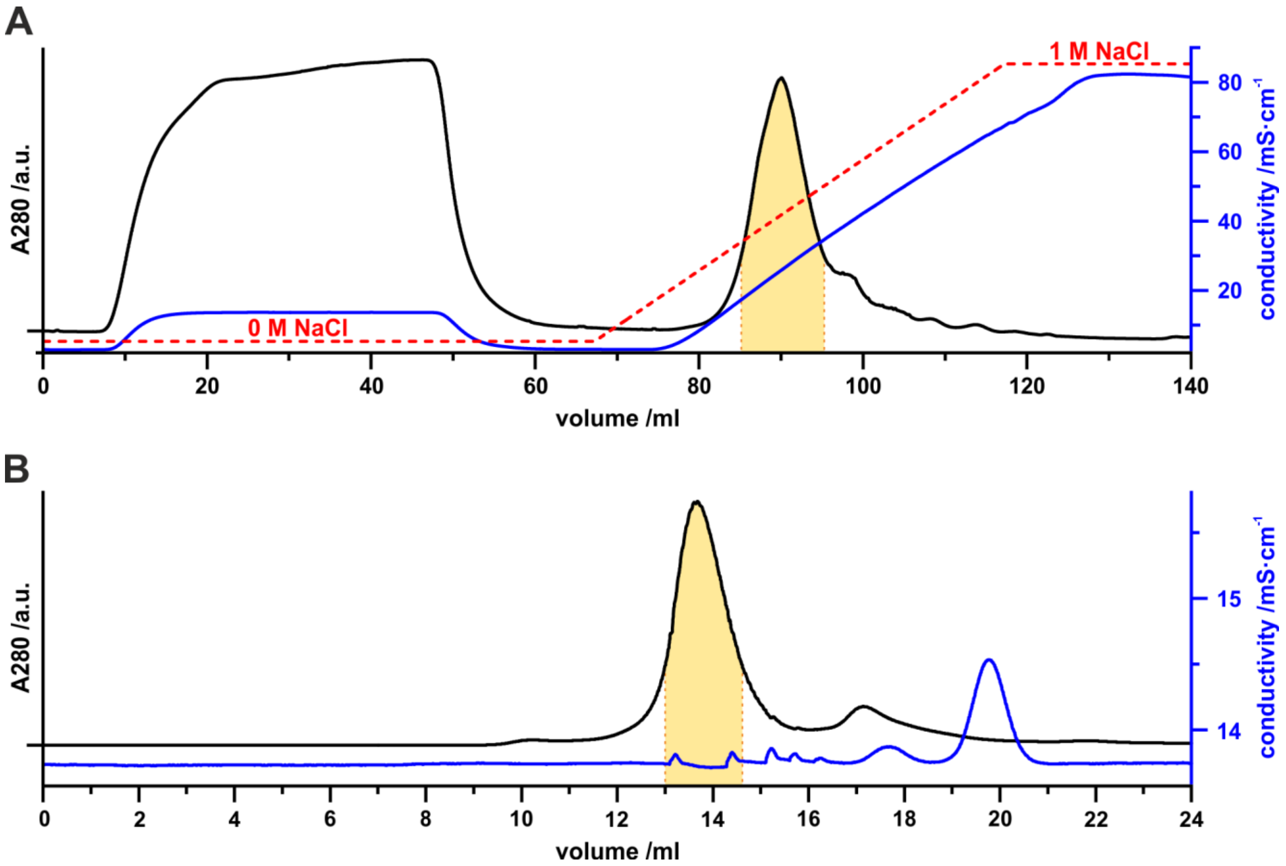

Supplementary Figure 1 Purification of IL-4 proteins refolded from *E. coli* inclusion bodies. (A) Fractionation of refolded protein by cation exchange chromatography. **(B)** Pooled fractions (yellow area highlighted in A) were further purified by size exclusion chromatography to remove a minor fraction of aggregated protein as well as low molecular weight contaminants. The main peak pooled fractions (highlighted in yellow) confirmed folding into globular, monomeric proteins. Representative chromatograms showing purification of KFR_D.

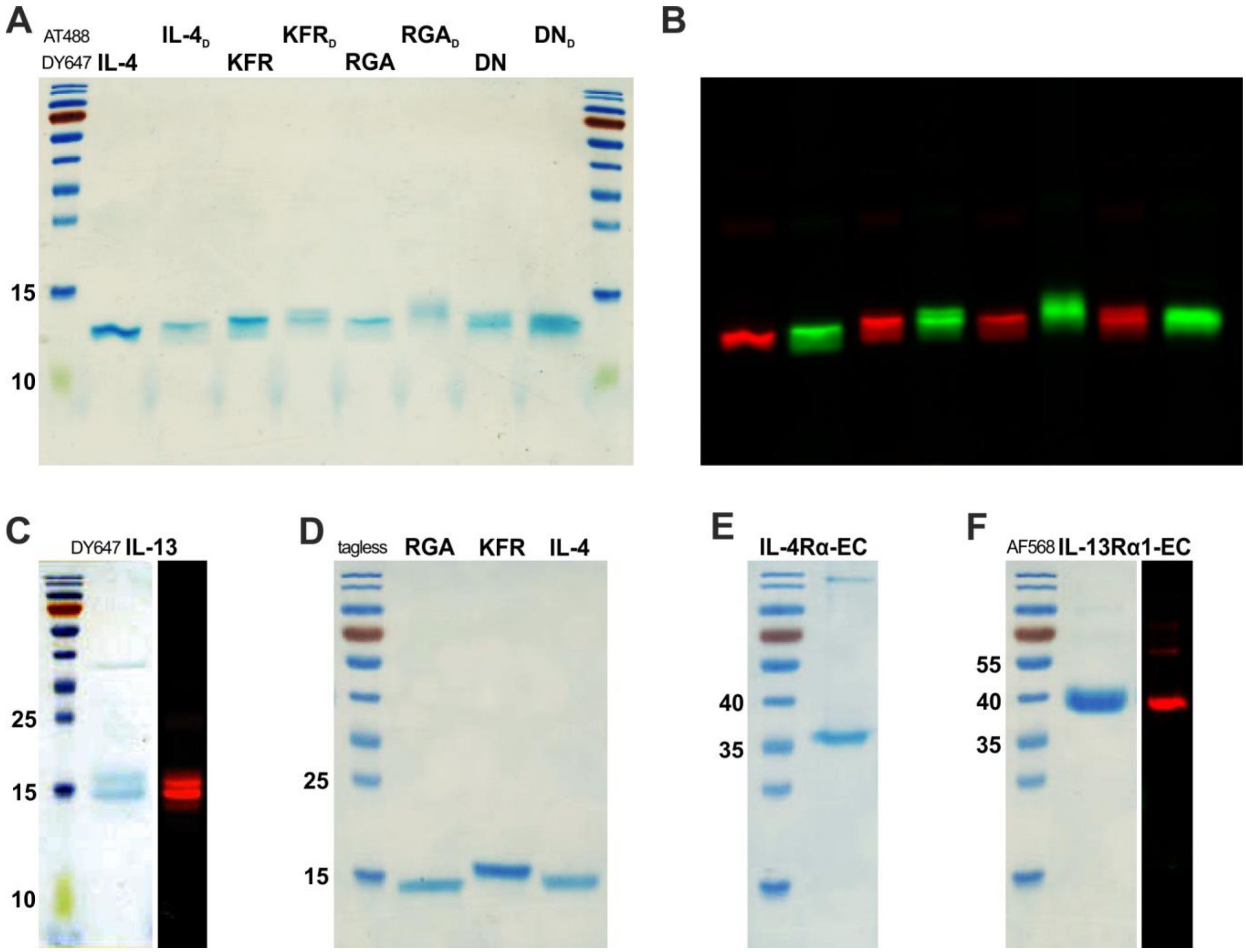

Supplementary Figure 2 Quality control of purified and labeled proteins by SDS-PAGE. (A, B) Comparison of all IL-4 variants after purification and site-specific fluorescence labeling using **(A)** Coomassie staining and **(B)** fluorescence detection. **(C)** Same analysis of labeled IL-13 (produced in insect cells yielding inhomogeneous glycosylation). **(D)** Tagless IL-4 variants, **(E)** unlabeled IL-4Rα-EC and **(F)** fluorescently labeled IL-13Rα-EC.

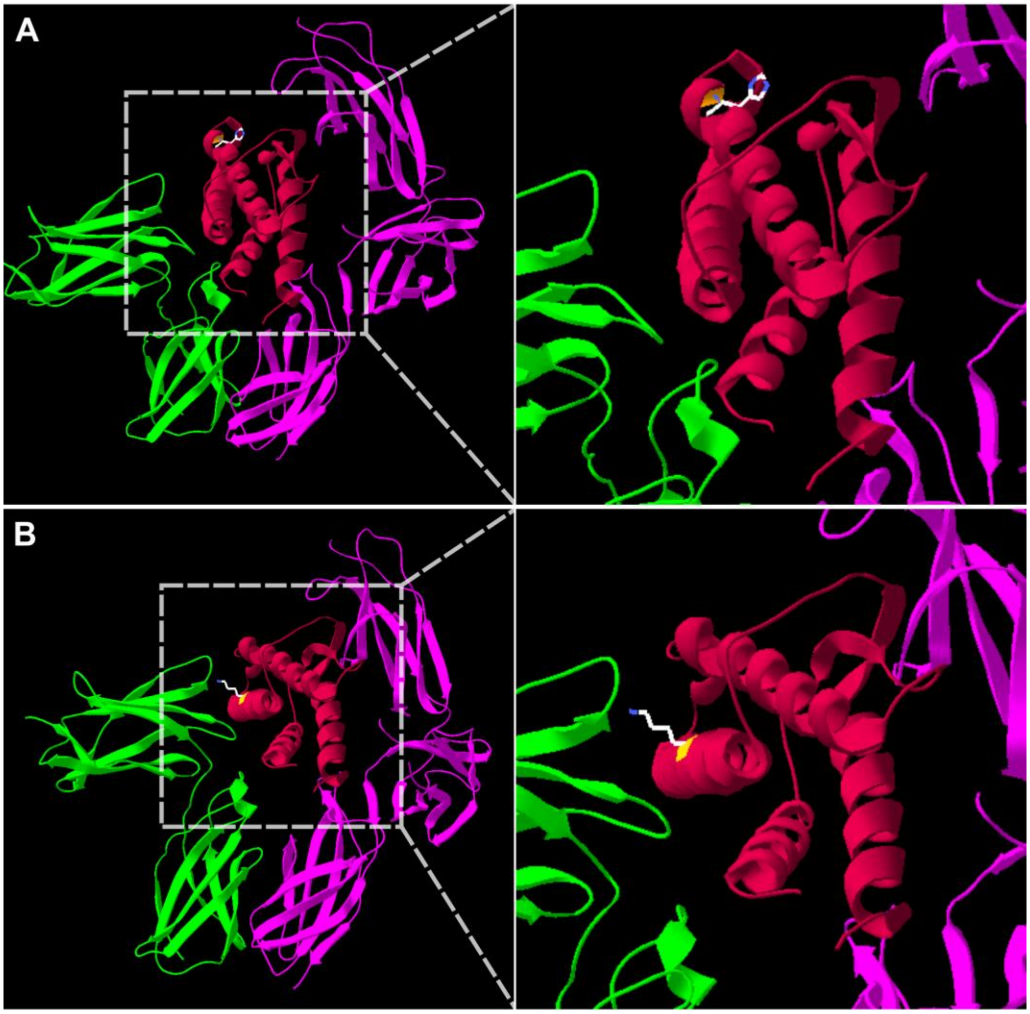

Supplementary Figure 3 Mutations introduced in IL-4 variants for TIRFS-RIf experiments. (A) H59Y: The Histidine residue at position 59 is changed into Tyrosine to eliminate non-specific binding to complexed Ni(II) ions. The mutated amino acid side-chain is oriented in direction of the protein core and doesn't affect receptor binding properties or activity of the ligand. **(B)** K84D: The Lysine residue at position 84 is replaced by an Aspartate, which increases the dissociation rate constant of the ligand-receptor complex to a similar level as of the IL-13/IL-13Rα1 interaction and prevents mass transport-limited association as well as strong rebinding during dissociation. As this mutation only changes the binding interface of the interaction with IL-4Rα while the IL-13Rα1 binding interface is unaltered, the interaction with IL-13Rα1 is not affected by this mutation.

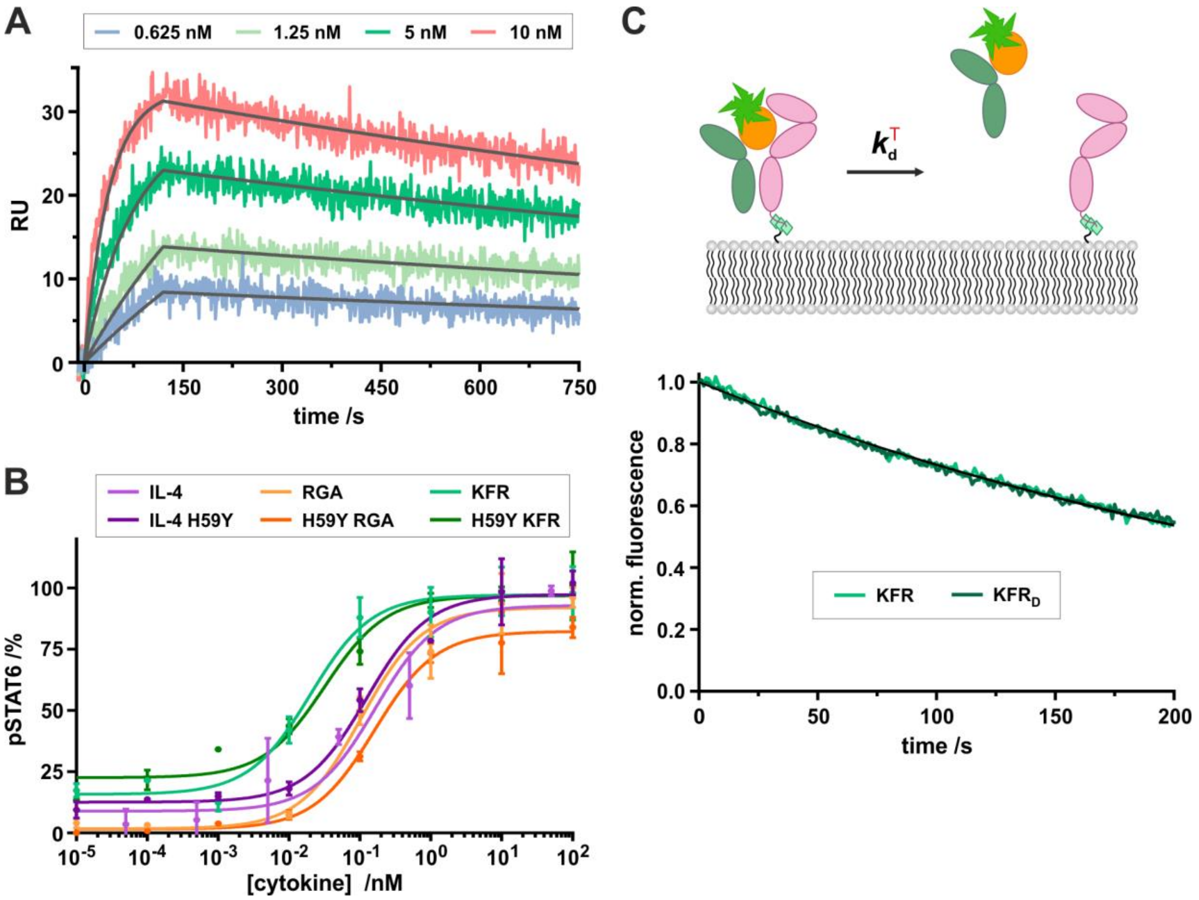

Supplementary Figure 4 Receptor interaction properties and functional integrity of IL-4 variants. (A) SPR measurements of IL-4Rα-EC binding at different concentrations to IL-4 (H59Y) immobilized on a Neutravidin chip via a biotinylated N-terminal ybbR-tag. Kinetic rate constants obtained by these measurements are similar to those of the interaction with IL-4 wt (see Supplementary Table 1). **(B)** pSTAT6 dose-response curves comparing the activity of IL-4 variants with and without H59Y mutation. Data points represent mean ± s.d. of three independent experiments. **(C)** Normalized dissociation of KFR and KFR_D variants (100 nM) in complex with tagless IL-4Rα-EC (1 µM) from surface-tethered IL-13Rα1-EC (5 fmol mm^{-2}). Overlapping curves confirm that the interaction with IL-13Rα1 is not affected by the K84D mutation.

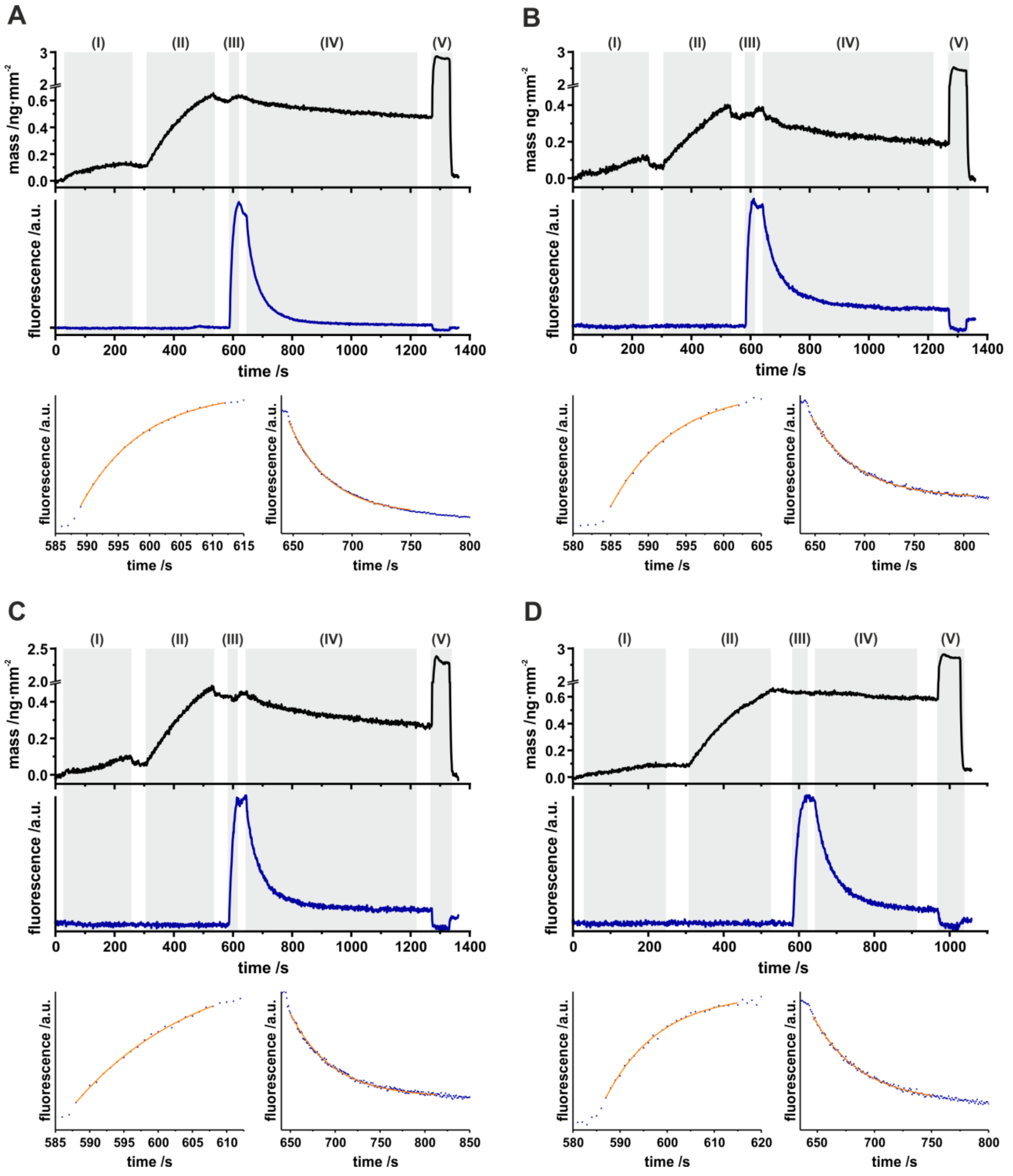

Supplementary Figure 5 **Quantification of binary complex interaction kinetics** of **(A)** ^{AT488}IL-4$_D$, **(B)** ^{AT488}KFR$_D$, **(C)** ^{AT488}RGA$_D$ binding to IL-4Rα-EC and of **(D)** ^{OG488}IL-13 binding to IL-13Rα1-EC by simultaneous TIRFS-RIf detection. Corresponding fits of association and dissociation phases below TIRFS-RIf traces. Experimental procedure on solid-supported membranes doped with tris-NTA lipids: (I) Tethering of IL-4Rα-EC (75 nM) or IL-13Rα1-EC (75 nM), respectively, and (II) MBP-H10 (2 µM) for blocking of excess chelator lipids, (III) followed by injection of the respective ligand (100 nM), (IV) a 600 s dissociation phase and (V) a final Imidazole wash. Depicted curves show representative single experiments.

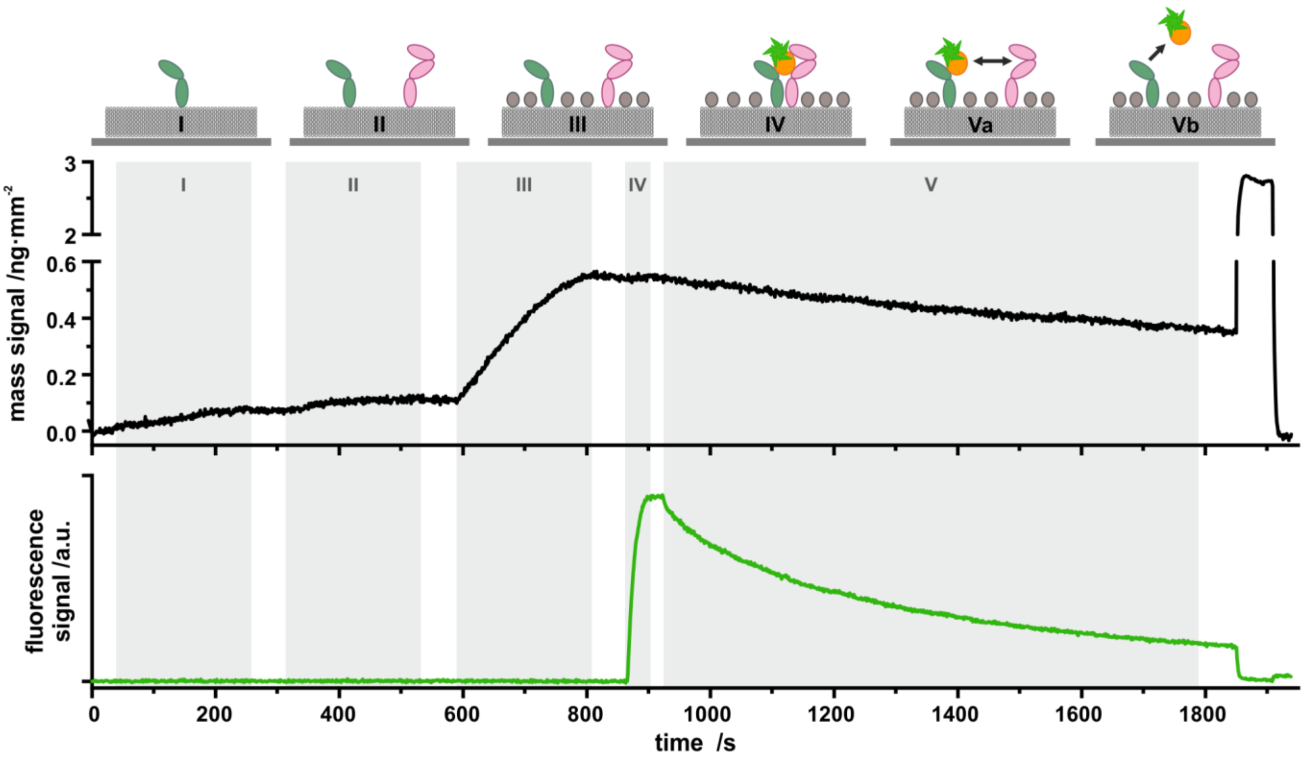

Supplementary Figure 6 Quantification of the ternary complex 2D equilibrium by simultaneous TIRFS-RIf detection. Top: cartoon of the assay: after sequentially tethering IL-4Rα-EC (I), IL-13Rα1-EC (II) and MBP (III) onto solid-supported membranes doped with tris-NTA lipids, the equilibrium between binary and ternary complexes (IV-Va) is probed by monitoring the dissociation of fluorescently labeled IL-4 variants and IL-13 (Vb). Bottom: typical assay including (I) sequential tethering of IL-4Rα-EC (75 nM), (II) IL-13Rα1-EC (100 nM) and (III) MBP-H10 (2 µM), (IV) injecting ^{AT488}IL-4_D (100 nM) and (V) rinsing for 900 s. Depicted curves show a representative single experiment.

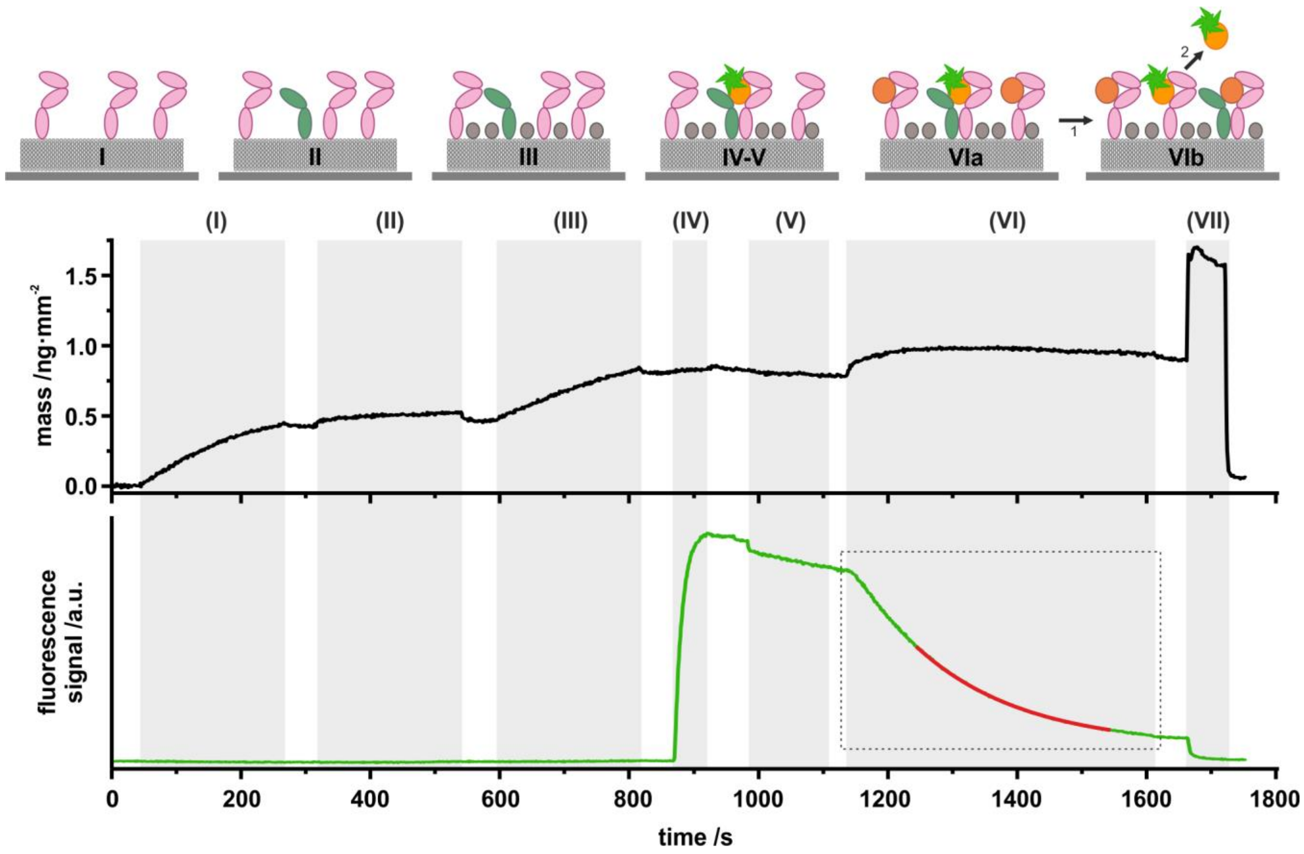

Supplementary Figure 7 Ligand chasing confirms formation of dynamic ternary complexes. After tethering (I) a large excess of IL-13Rα1-EC (300 nM) over (II) IL-4Rα-EC (20 nM) and (III) blocking with MBP-H10 (1 µM), (IV) ^{AT488}IL-4$_D$ was loaded under conditions saturating IL-4Rα-EC (100 nM). Then, after (V) rinsing for monitoring spontaneous dissociation of ^{AT488}IL-4$_D$, (VI) excess IL-13Rα1-EC was saturated by injecting the high affinity IL-13 mutant A11 [7] (0.5 µM). IL-13Rα1-EC/IL-13 A11 competes with IL-13Rα1-EC/^{AT488}IL-4$_D$ (step1), followed by rapid dissociation of ^{AT488}IL-4$_D$ in binary complex with IL-13Rα1-EC (step 2) and exchange by IL-13 A11 (not shown). As step 1 is rate-limiting, this assay probes the 2D dissociation rate constant of IL-4Rα-EC with ^{AT488}IL-4$_D$/IL-13Rα1-EC, which was determined by an exponential fit (red curve, rate constant $6.1\pm0.1\cdot10^{-3}$ s). After the experiment, all proteins were removed by injecting imidazole (500 mM) (VI). Depicted curves show a representative single experiment.

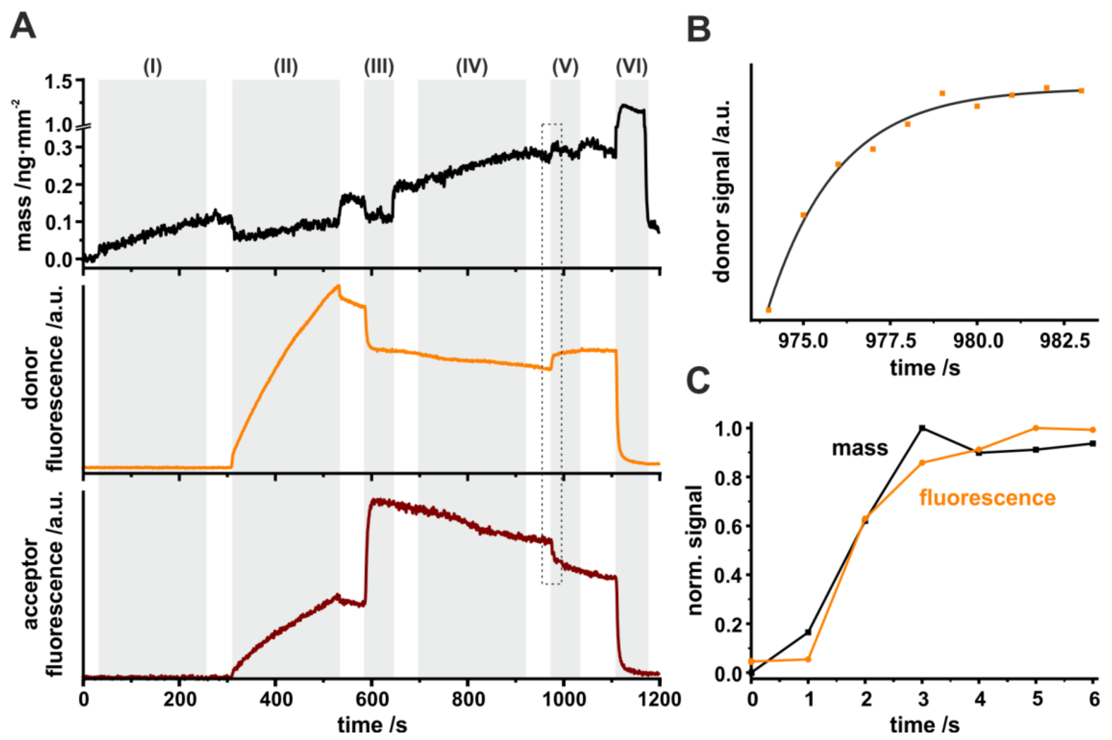

Supplementary Figure 8 Quantification of the 2D dissociation kinetics of RGA-induced ternary complexes on SSMs by **(A)** simultaneous TIRFS-RIf detection and utilizing FRET between the donor-labeled receptor (orange curve) and the acceptor-labeled ligand (red curve). The donor fluorescence is decreased after ligand injection (III) because of FRET to the ligand associated acceptor in ternary complexes. Chasing with excess unlabeled high-affinity ligand (V) leads to an exchange of ligands in ternary complexes and therefore a donor fluorescence recovery, which rate corresponds to the ternary complex dissociation rate if chasing is fast enough. **(B)** Fit of the donor fluorescence signal recovery and **(C)** normalized comparison of the donor fluorescence and the mass signal. Latter reveals that chasing is limited by mass transport/association kinetics and therefore the ternary complex dissociation rate for RGA is not measurable with this approach. Experimental procedure on solid-supported membranes doped with tris-NTA lipids: (I) Tethering of IL-4Rα-EC (100 nM) and (II) ^{AF568}IL-13Rα1-EC (150 nM), (III) followed by injection of ^{DY647}RGA (100 nM) and (IV) additional tethering of an excess of IL-4Rα-EC (1 µM). (V) Chasing by fast injection of 1 µM IL-4 (dashed rectangle). (VI) Final imidazole wash for surface regeneration. Depicted curves show a representative single experiment.

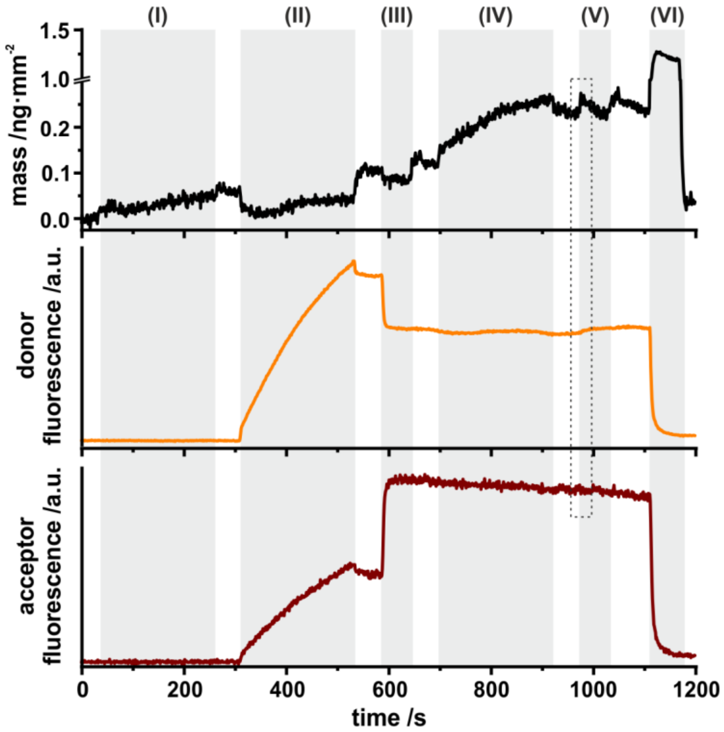

Supplementary Figure 9 Quantification of the 2D dissociation kinetics of KFR-induced ternary complexes on SSMs by simultaneous TIRFS-RIf detection and utilizing FRET between the donor-labeled receptor (orange curve) and the acceptor-labeled ligand (red curve). The donor fluorescence is decreased after ligand injection (III) because of FRET to the ligand associated acceptor in ternary complexes. Chasing with excess unlabeled high-affinity ligand (V) leads to an exchange of ligands in ternary complexes and therefore a donor fluorescence recovery, which rate corresponds to the ternary complex dissociation rate if chasing is fast enough. No donor recovery (i.e. ligand exchange) can be observed for KFR because of a very low (and with this approach not measurable) dissociation rate leading to highly stable complexes. Experimental procedure on solid-supported membranes doped with tris-NTA lipids: (I) Tethering of IL-4Rα-EC (100 nM) and (II) ^{AF568}IL-13Rα1-EC (150 nM), (III) followed by injection of ^{DY647}KFR (100 nM) and (IV) additional tethering of an excess of IL-4Rα-EC (1 µM). (V) Chasing by fast injection of IL-4 (1 µM, dashed rectangle). (VI) Final imidazole wash for surface regeneration. Depicted curves show a representative single experiment.

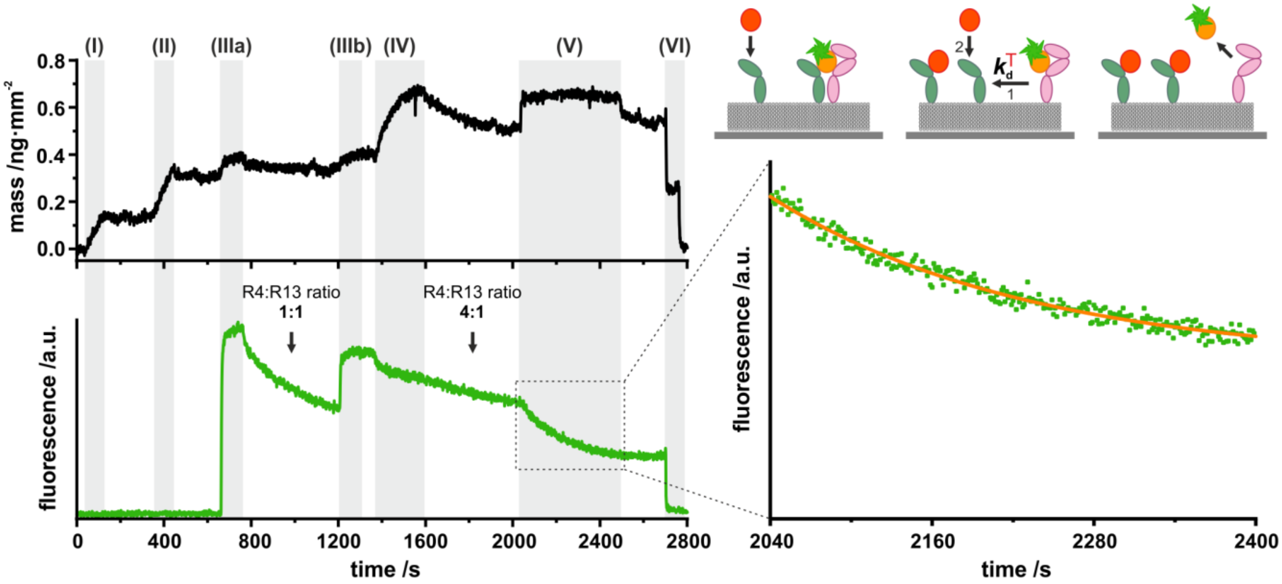

Supplementary Figure 10 Quantification of the 2D dissociation kinetics of IL-13-induced ternary complexes by simultaneous TIRFS-RIf detection. The cartoon shows the mechanism behind this chasing experiment. During chasing each IL-4Rα-EC that dissociates from a ternary complex (step 1) readily binds an IL-4 ligand (step 2) with sub-nanomolar affinity and is not available for ternary complex reassembly. Binary ^{OG488}IL-13/IL-13Rα1-EC complexes dissociate faster than ternary complexes, whose dissociation rate k_d^T is the limiting step in this experiment and is obtained by fitting the decreasing fluorescence signal (left figure, dashed rectangle) with an exponential function (right figure). Note that in the depicted experiment the ligand dissociation at increased surface concentration of second receptor chain IL-4Rα-EC (4:1 ratio) is significantly lower (second dissociation phase) compared to the dissociation at stoichiometrically equal amounts of receptors (first dissociation phase, cf. Figure 1C). Experimental procedure on solid-supported membranes doped with tris-NTA lipids: (I) Tethering of IL-13Rα1-EC (100 nM) and (II) IL-4Rα-EC (100 nM), (IIIa and IIIb) followed by two injections of ^{OG488}IL-13 (100 nM) and (IV) additional tethering of an excess of IL-4Rα-EC (1 µM). (V) Chasing by injection of IL-4 (1 µM). (VI) Final imidazole wash for surface regeneration.

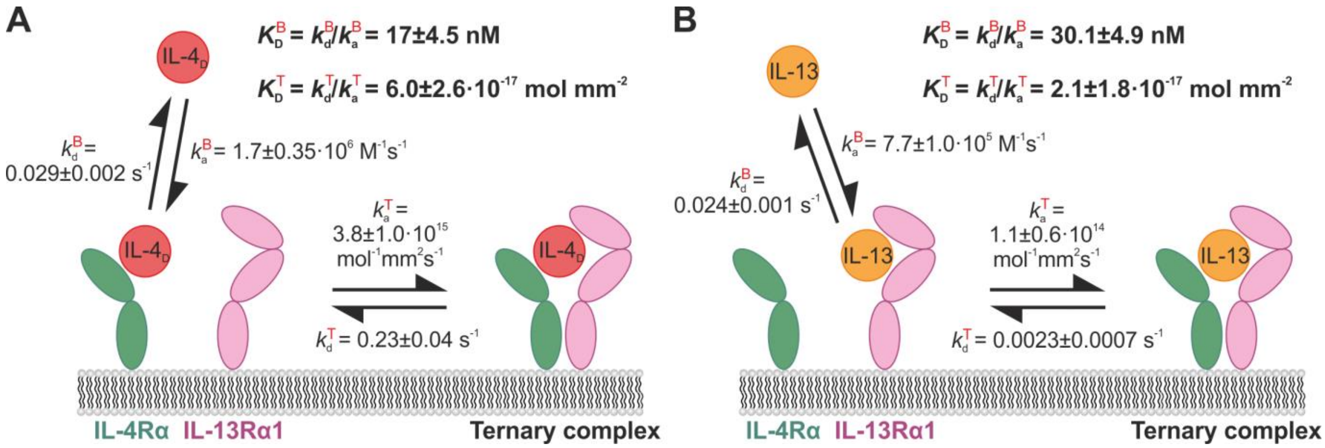

Supplementary Figure 11 Fully parameterized two-step association and dissociation model of the type-II Interleukin-4 receptor system. Ternary complex dynamics are not altered by H59Y K84D mutations of IL-4, while binary complex affinity is 100-fold lower compared to wildtype IL-4.

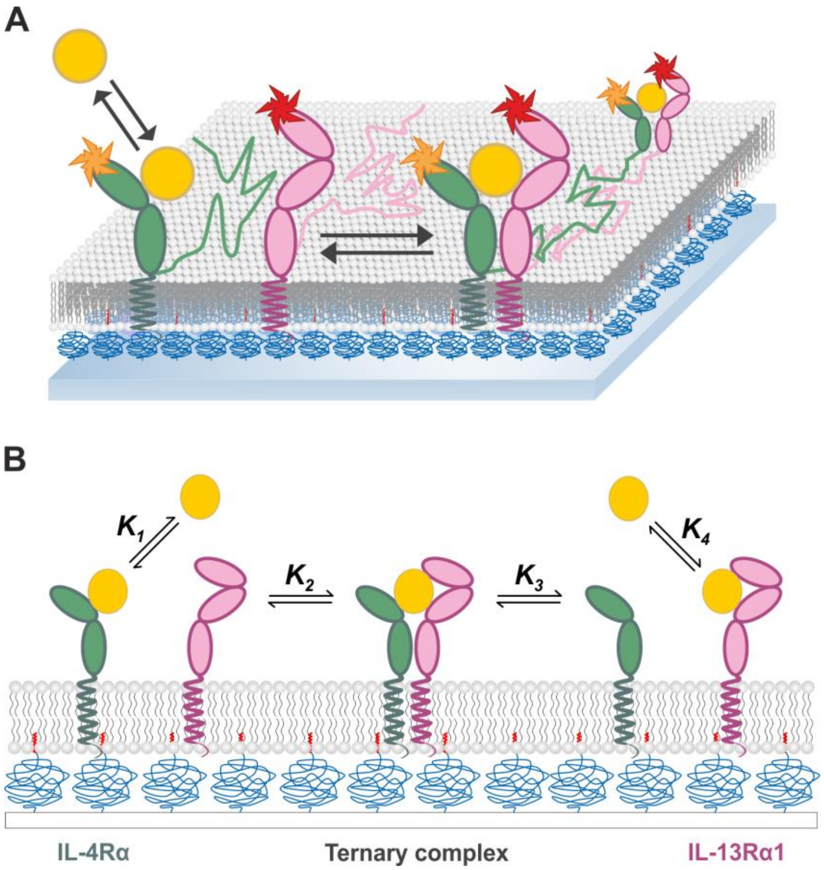

Supplementary Figure 12 Ligand-induced dimerization of transmembrane receptors reconstituted into polymer supported membranes. SNAPf-IL-4Rα-TM and SNAPf-IL-13Rα1-TM were expressed in HEK 293T cells and labeled with BG-547 and BG-647, respectively. **(A)** After affinity purification, receptors were reconstituted into 98/2 mol% DOPC/DOPS polymer supported membranes and their dimerization was quantified by single molecule co-localization. **(B)** Ligand-induced dimerization of IL-4Rα and IL-13Rα1 is determined by four equilibria. K_1 and K_4 are 3D equilibrium dissociation constants (unit: [mol l^{-1}]), K_2 (corresponding to K_D^T in Figure 1A) and K_3 are 2D equilibrium dissociation constants (unit: [molecules μm^{-2}]).

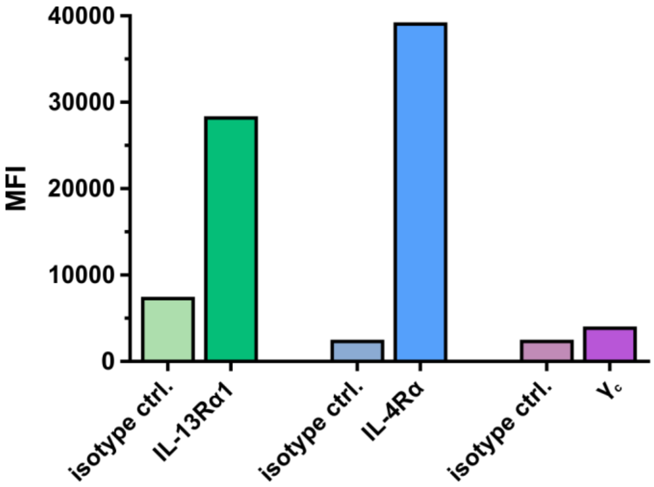

Supplementary Figure 13 Expression of the IL-4 receptor subunits on the surface of HeLa cells. Cells were stained with fluorescently labeled antibodies against IL-13Rα1, IL-4Rα and γ_c, respectively. Expression levels were assessed by flow cytometry and the mean fluorescence intensity (MFI) was determined from three independent experiments. Appropriate isotype controls served as negative control.

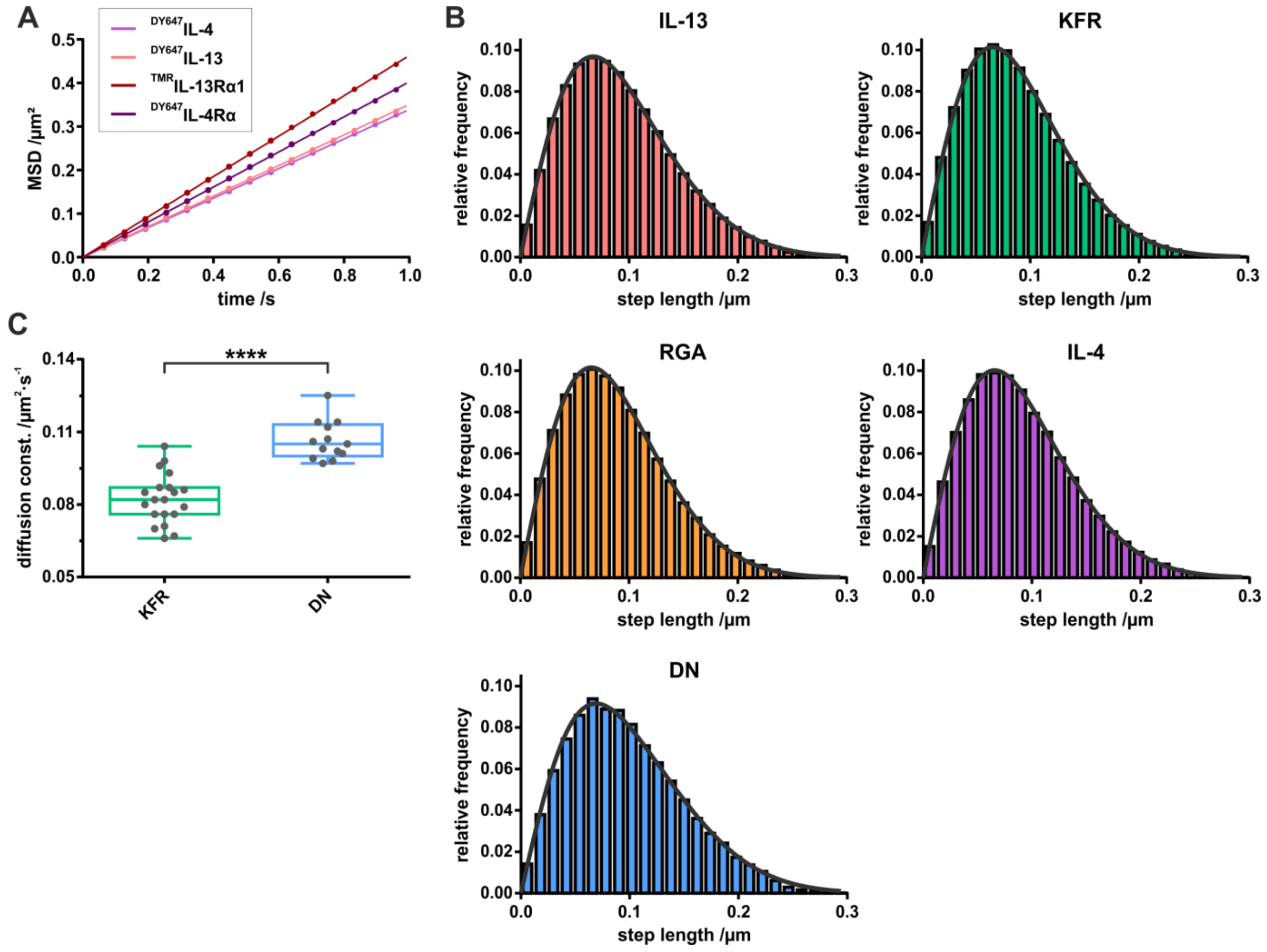

Supplementary Figure 14 Diffusion properties of IL-13Rα1 and IL-4Rα. (A) Mean square displacement (MSD) plot of ^{TMR}IL-13Rα1 (w/o ligand)**,** ^{DY647}IL-4Rα (w/o ligand)**,** ^{DY647}IL-13 and ^{DY647}IL-4, and **(B)** fitted (black lines) step length histograms (time lapse 32 ms, 1 frame) of DY647-labeled IL-4 variants and ^{DY647}IL-13 bound to the respective cell surface receptor on HeLa cells. Data are based on trajectories with min. length of 150 frames, pooled from experiments of at least 10 cells. **(C)** Comparison of the diffusion constants obtained for KFR and DN, respectively, as determined by MSD-analysis of 5 consecutive steps with a time lapse of 32 ms. Each data point in the box plot corresponds to a diffusion constant determined from all trajectories of a single cell. Statistical analysis by Student´s t-test (****, $p < 0.0001$).

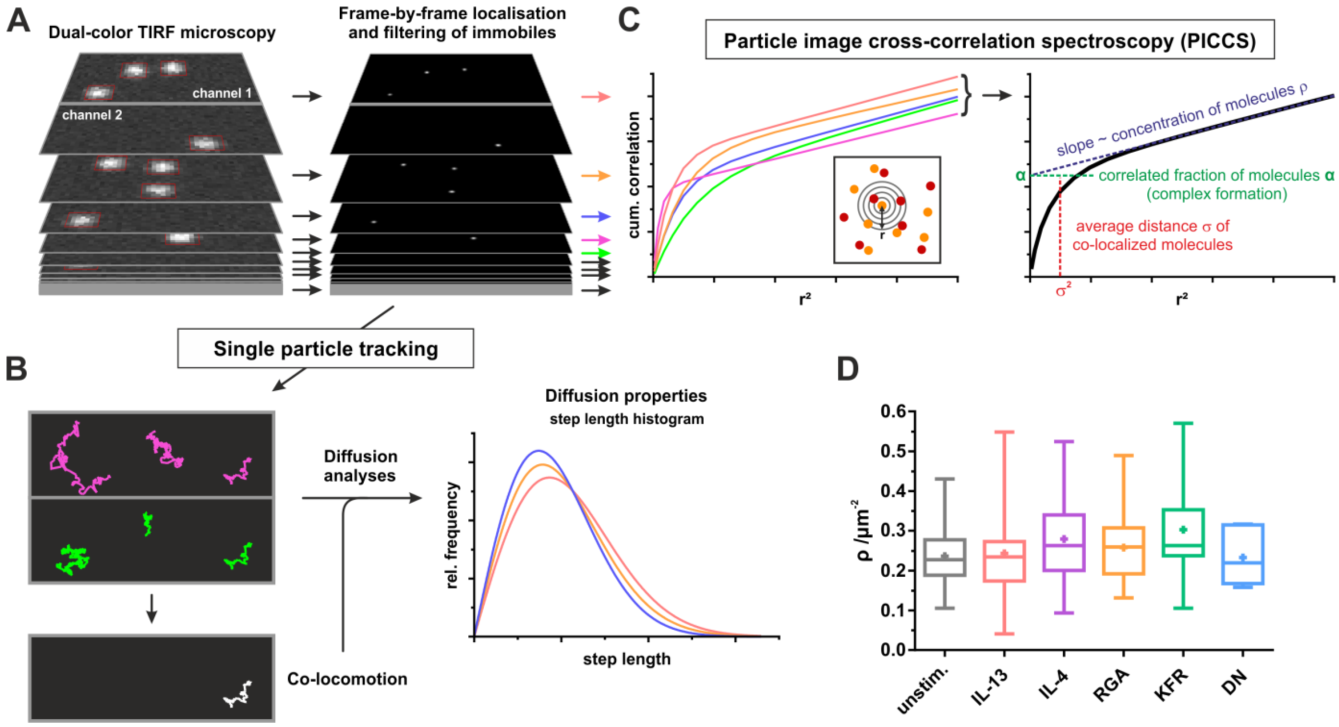

Supplementary Figure 15 Spatiotemporal correlation analyses of dual color imaging data. (A-C) Work flow including single molecule localization (A), co-locomotion and diffusion analyses (B) as well spatial correlation by PICCS (C). **(A)** Individual molecules detected in each frame are localized by fitting with a two-dimensional Gaussian function. Immobile molecules identified by spatiotemporal cluster analysis are removed from the data set prior to further data processing. **(B)** Tracking of molecules in each channel separately (pink and green trajectories) and co-tracking for identifying receptor heterodimers (white trajectories). The mobility of individual receptor subunits and complexes is analyzed by a histogram of the step lengths extracted from the trajectories. **(C)** The relative amount of ternary complexes is quantified by particle image cross-correlation spectroscopy (PICCS) of the localized molecules in both channels. The averaged PICCS curve is fitted to yield the correlated fraction α as a measure of receptor dimerization, the correlation length σ as measure of the distance within dimers and the linear slope ρ as a measure of molecule density. **(D)** Comparison of ^{DY647}IL-4Rα receptor densities ρ in dual-color single molecule imaging experiments as obtained from PICCS analysis.

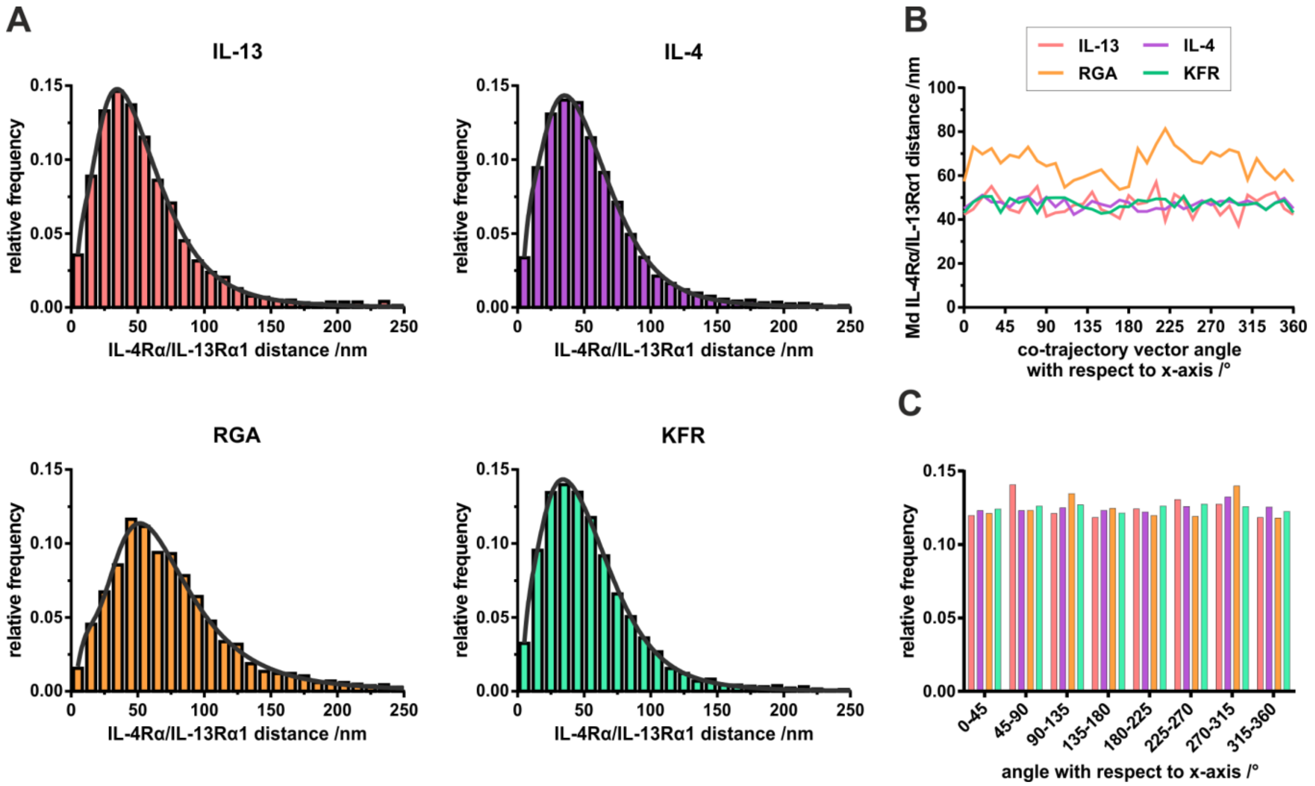

Supplementary Figure 16 **DY647IL-4Rα/TMRIL-13Rα1 distance during co-locomotion.** **(A)** Distance distributions of receptor subunits in ternary complexes induced by IL-4 variants and IL-13 show a significant shift to longer distances for RGA, **(B)** which is independent of the direction of movement, while the **(C)** distribution of movement directions is equal for all ligands. Thus, an experimental artifact due to an inaccurate spectral channel alignment can be excluded, as this would result into a shifted distance distribution for RGA into one predominant direction.

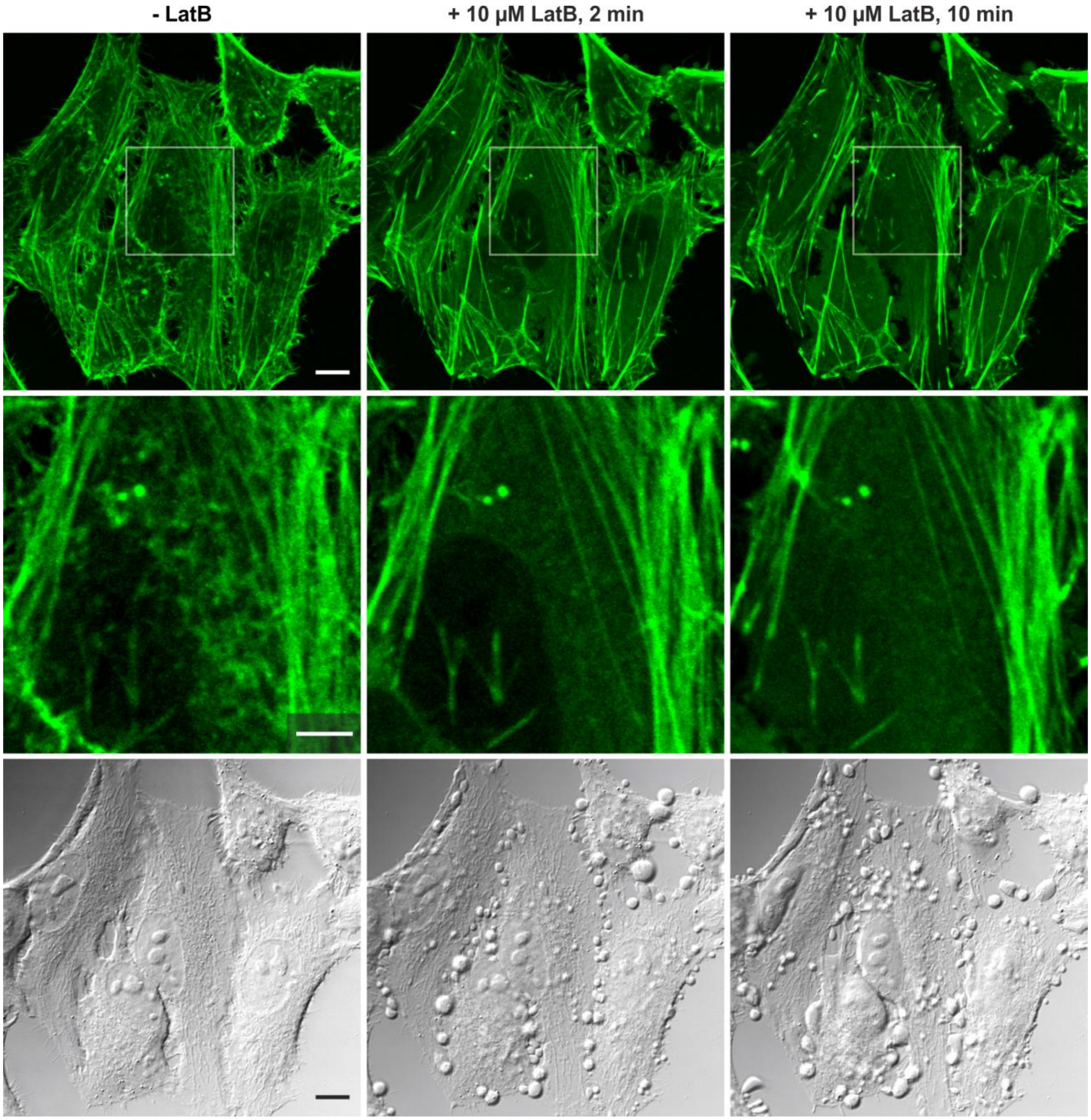

Supplementary Figure 17 Effects of Latrunculin B treatment on the actin cytoskeleton. HeLa cells stably expressing Lifeact-mEGFP to stain the actin cytoskeleton [8] were imaged by confocal fluorescence microscopy before and after addition of 10 µM Latrunculin B (LatB). The fine-structures of actin filaments almost completely disappeared already after 2 minutes of drug treatment with only a minor change after another 8 min. Magnified regions (middle row) are marked in white. Corresponding DIC images (bottom) reveal the characteristic formation of membrane blebs due to the LatB-induced detachment and loss of the cortical membrane cytoskeleton. Scale bars: 10 µm (top and bottom row), 5 µm (middle row).

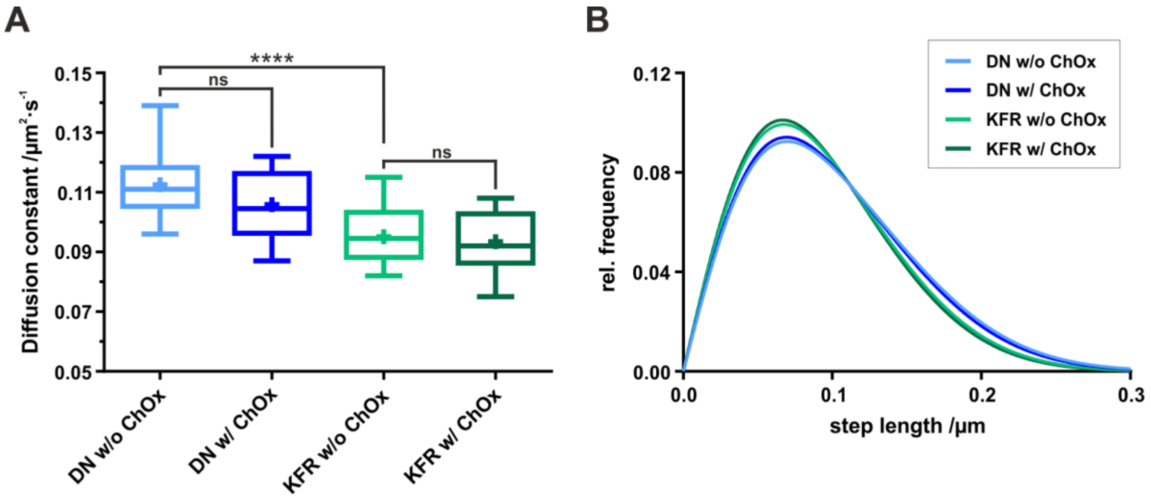

Supplementary Figure 18 Effects of cholesterol depletion on receptor mobility. (A) Comparison of diffusion constants of IL-4Rα labeled with ^{DY647}DN and ^{DY647}KFR, respectively, obtained by MSD-analysis (5 steps) of the trajectories (min. length: 30 frames) of different cells (n=20) with and without cholesterol depletion by treatment with 20 U cholesterol oxidase (ChOx) for 30 min at 37°C. Statistical analysis by Student´s t-test (****, $p < 0.0001$; ns, not significant). **(B)** Step-length histograms of pooled data from A.

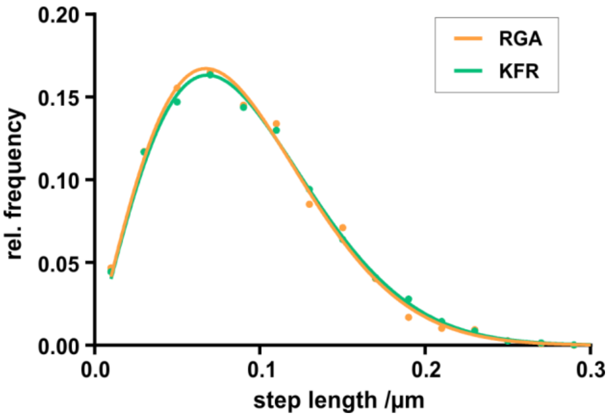

Supplementary Figure 19 Diffusion properties of KFR- and RGA-induced dimers in presence of LatB. Step-length distributions (time lapse 32 ms, 1 frame) of co-trajectories, based on two components corresponding to a slow and a fast mobile fraction. Depicted curves are based on pooled co-trajectory-data (min. length 30 frames) of *n*>17 cells trajectories.

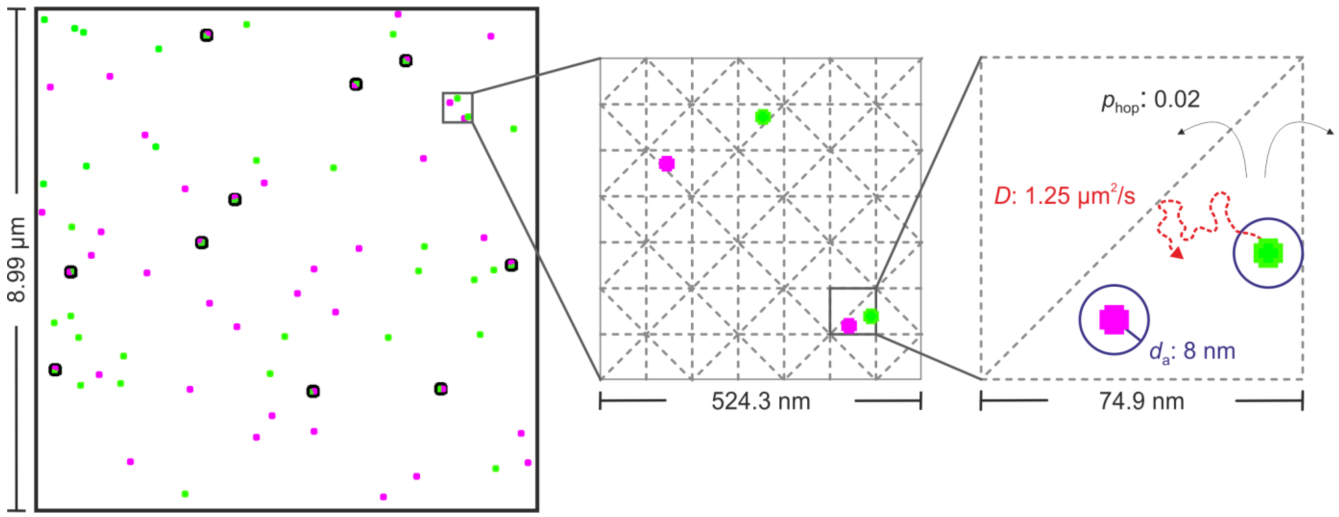

Supplementary Figure 20 Simulation of spatiotemporal receptor dynamics based on a simple MSK hop-diffusion model. Illustration of a representative state during a simulation run. Dots indicate binary complexes of ligand and high affinity ligand-binding receptor subunit (green), and accessory receptor subunits (magenta). Ternary complexes are marked with black circles. Receptor molecules are diffusing (D=1.25 µm^2 s^{-1}) inside membrane compartments (triangles with 74.9 nm edge length) and can pass boundaries (dashed grey lines) with possibility p_{hop} (0.02 s^{-1}). Interactions may occur if two molecules come into closer proximity than an association distance threshold d_{a} (8 nm). Association and dissociation events occur in dependence of fixed possibilities that are estimated from spatial correlation analyses in living cells (p_{a}) or calculated from the molecular kinetic rate constants measured in *vitro* (p_{d}).

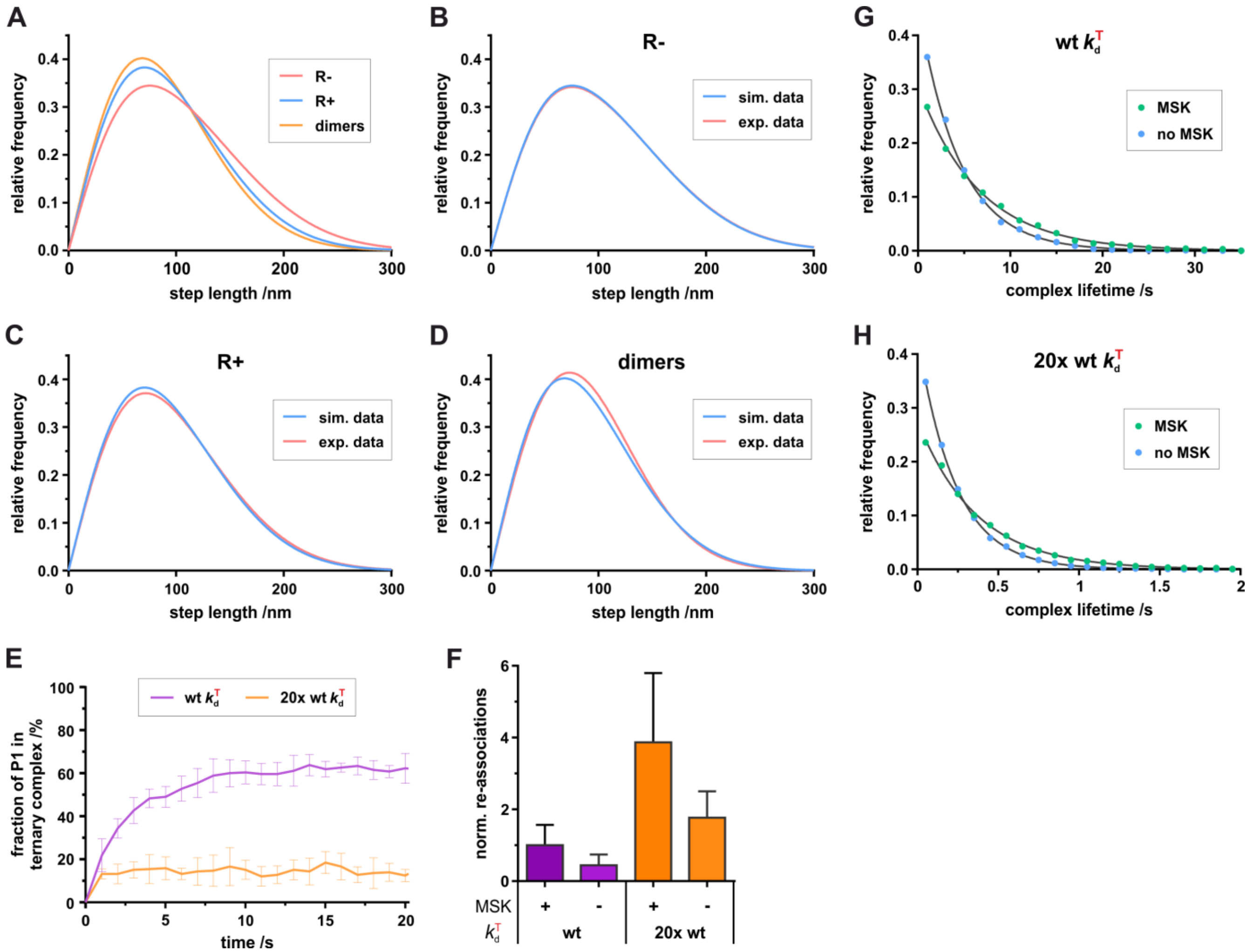

Supplementary Figure 21 Simulation of receptor diffusion and complex dynamics by DAIDS. (A) Diffusion properties of simulated receptors with (blue, wt k_d^T) and without (red) ligand, and in ternary complexes (orange) presented as fitted step-length distributions (time lapse 32 ms, 1 frame), based on two components corresponding to a slow and a fast mobile fraction (cf. Fig. 3C). A comparison of experimentally obtained (^{DY647}IL-4Rα in B+C, ^{DY647}IL-4Rα/^{TMR}IL-13Rα1 in D) and simulation-derived data shows that **(B)** the distributions perfectly match for unstimulated receptors and that a high degree of similarity is present for **(C)** ligand exposed receptors and **(D)** for receptors in ternary complexes. **(E)** Although the receptor subunits are randomly distributed at the beginning of each simulation run, a dynamic equilibrium between binary and ternary complexes is established in less than 2 (20x wt k_d^T) to 10 (wt k_d^T) seconds while up to 60% of receptors are part of a ternary complex (wt k_d^T). **(F)** Without the simulated membrane confinement by the MSK, the number of contemporary re-associations per particle and time is reduced by more than 50%. Furthermore, the amount of re-associations increases with the probability of the ternary complex to dissociate (i.e. increases with higher k_d^T). **(G-H)** Frequency distributions of ternary complex lifetimes (measured as the time both receptor subunits reside in the same compartment after undergoing at least one association event) fitted with an exponential decay function: wt k_d^T: t=4460±60 ms (no MSK), t=6560±88 ms (MSK). 20x wt k_d^T: t=233±1 ms (no MSK), t=363±5 ms (MSK). Data in A-E from 5 independent simulation runs (parameter-set A) and in F-H from 10 independent simulation runs for each ligand scenario (parameter-set B).

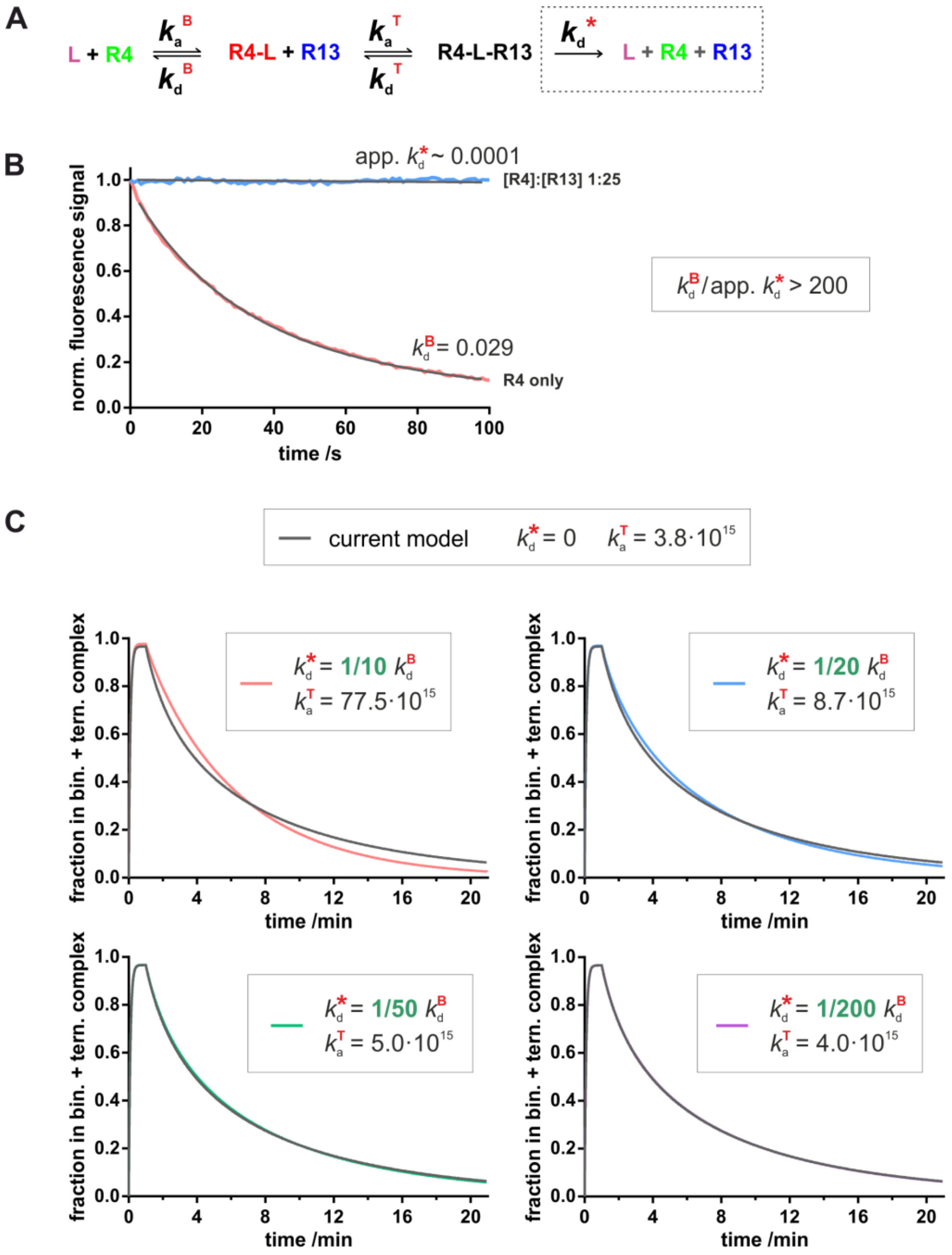

D

k_d*	0	1/10	1/20	1/50	1/100	1/200	x k_d^B
k_a^T	3,8	77,5	8,7	5,0	4,3	4,0	x 10^15
DEV	-	20,40	2,30	1,32	1,14	1,07	-fold
	-	2040	230	132	114	107	%

Supplementary Figure 22 Validation of two-step assembly/disassembly model. (A) Alternative model including direct dissociation from ternary complexes (dotted rectangle). **(B)** Experimental data with a high surface concentration of IL-13Rα1 reveals more than 200-fold faster dissociation of ^{AT488}IL-4_D from binary than from ternary complexes. **(C, D)** Simulations assuming different contributions of direct dissociation (quantified relative to the binary (k_d) and the k_a^T obtained by fitting the simplified model. Under the experimentally relevant conditions (highlighted in red) <10% bias compared to the ideal value (no direct dissociation) was observed.

Supplementary Table 1 Mutations and tagging of the recombinant proteins used in this study.

Interleukin variant	Abbreviation	Used for[a]	Mutations	Expressed in
ybbR-tagged				
IL-13 wt	IL-13	A, C	-	*Hi5*
IL-4 H59Y	IL-4	A, C	H59Y	*E. coli*
IL-4 H59Y KFR	KFR	A, C	H59Y, R121K, Y124F, S125R	*E. coli*
IL-4 H59Y RGA	RGA	A, C	H59Y, K117R, T118V, R121Q, E122S, Y124W, S125F, S128G, S129A	*E. coli*
IL-4 H59Y DN	DN	A, B	H59Y, R121D, Y124D	*E. coli*
IL-4 H59Y K84D	IL-4_D	C	H59Y K84D	*E. coli*
IL-4 H59Y K84D KFR	KFR_D	C	H59Y, K84D, R121K, Y124F, S125R	*E. coli*
IL-4 H59Y K84D RGA	RGA_D	C	H59Y, K84D, K117R, T118V, R121Q, E122S, Y124W, S125F, S128G, S129A	*E. coli*
IL-4 H59Y K84D DN	DN_D	C	H59Y, K84D, R121D, Y124D	*E. coli*
IL-13Rα1-EC-H10	IL-13Rα1-EC	C	ECD only (residues 29-344)	*Hi5*
IL-4Rα-EC-H10	IL-4Rα-EC	C	ECD only (residues 27-232)	*Hi5*
tagless				
IL-13 wt	IL-13	B, D	-	*Hi5*
IL-4 wt	IL-4	B, D	-	*Hi5*
IL-4 KFR [4]	KFR	B, D	R121K, Y124F, S125R	*Hi5*
IL-4 RGA [4]	RGA	B, D	K117R, T118V, R121Q, E122S, Y124W, S125F, S128G, S129A	*Hi5*

[a] A: Ligand binding experiments by TIRFM; B: Transmembrane receptor dimerization experiments in PSM and living cells by TIRFM; C: in vitro kinetic studies by TIRFS-RIf detection; D: Activity assays by phospho-flow cytometry.

Supplementary Table 2 Conjugates used for labeling of recombinant proteins used in this study.

Protein	Dye	Designation
ybbR-IL-13 wt	CoA-DY647	^{DY647}IL-13
ybbR-IL-13 wt	CoA-OregonGreen 488	^{OG488}IL-13
ybbR-IL-4 H59Y	CoA-DY647	^{DY647}IL-4
ybbR-IL-4 H59Y KFR	CoA-DY647	^{DY647}KFR
ybbR-IL-4 H59Y RGA	CoA-DY647	^{DY647}RGA
ybbR-IL-4 H59Y DN	CoA-DY647	^{DY647}DN
ybbR-IL-4 H59Y K84D	CoA-Atto 488	^{AT488}IL-4$_D$
ybbR-IL-4 H59Y K84D KFR	CoA-Atto 488	^{AT488}KFR$_D$
ybbR-IL-4 H59Y K84D RGA	CoA-Atto 488	^{AT488}RGA$_D$
ybbR-IL-4 H59Y K84D DN	CoA-Atto 488	^{AT488}DN$_D$
ybbR-IL-13Rα1-EC-H10	CoA-Alexa Fluor 568	^{AF568}IL-13Rα1-EC

Supplementary Table 3 Kinetic and equilibrium constants of binary ligand-receptor interactions.

Interacting moieties	k_a^B [M^{-1} s^{-1}]	k_d^B [s^{-1}]	K_D^B [nM]
IL-4$_D$ ↔ IL-4Rα	$(1.7±0.4)·10^6$	0.029±0.002	17.1±4.5
IL-13 ↔ IL-13Rα1	$(0.8±0.1)·10^6$	0.024±0.001	30.1±4.9
KFR$_D$ ↔ IL-4Rα	$(1.0±0.1)·10^6$	0.021±0.001	22.3±2.9
RGA$_D$ ↔ IL-4Rα	$(0.4±0.1)·10^6$	0.022±0.002	60.7±21.7
DN$_D$ ↔ IL-4Rα	$(0.3±0.1)·10^6$	0.024±0.004	83.7±42.0
IL-4 ↔ IL-4Rα	$(1.3±0.3)·10^7$	0.0013±0.0002	0.10±0.02[a]
IL-4 H59Y ↔ IL-4Rα	$(3.8±0.4)·10^7$	0.0019±0.0003	0.05±0.01
DN ↔ IL-4Rα	-	-	0.10±0.01[b]
IL-4 ↔ IL-13Rα1	-	-	2500[a]
IL-13 ↔ IL-4Rα	n.b.[c]	n.b.[c]	n.b.[c]

[a] from reference [5]
[b] from reference [6]
[c] no interaction detectable [5]

Supplementary Table 4 Comparison of interaction constants obtained by PICCS analyses.

Ligand	α [%][a]	σ [µm] [b]	ρ [µm^{-2}] [c]
unstimulated	1.1±0.6	0.14±0.08	0.24±0.09
DN	2.8±1.0	0.11±0.05	0.23±0.07
RGA	5.2±2.1	0.14±0.05	0.26±0.09
IL-13	7.4±3.2	0.07±0.03	0.24±0.10
KFR	8.5±2.7	0.09±0.03	0.30±0.11
IL-4	10.9±2.7	0.07±0.02	0.28±0.12
IL-4Rα + IL-4	28.4±5.5	0.07±0.01	0.40±0.10
IL-13Rα1 + IL-4	17.6±4.7	0.13±0.01	0.31±0.10
positive control	38.2±4.0	0.06±0.01	0.57±0.26

[a] Correlated fraction
[b] Correlation length
[c] Particle density

Supplementary Table 5 IL-4Rα/IL-13Rα1 distances determined from individual dimers induced by different ligands.

Distance fraction [a]	IL-4	KFR	RGA	IL-13
Fraction 1 [%]	35±2	34±2	0±2	30±2
Distance fraction 1 [nm]	30±1	30±1	30±1	30±1
Fraction 2 [%]	54±3	55±3	34±4	65±3
Distance fraction 2 [nm]	50±1	50±1	50±1	50±1
Fraction 3 [%]	11±5	10±5	66±6	5±5
Distance fraction 3 [nm]	70±1	70±1	70±1	70±1

[a] Fractions and standard deviations were fitted with a three component log-normal distribution with fraction distances fixed set to 30, 50 and 70 nm as determined by previous fits of the distributions with variable distances.

Supplementary Table 6 Sequences of primers used for cloning of vector constructs.

Gene [a]	Primer sequence (5'-3')
IL-4Rα for	GTGGCAAGCTCTAGATCTATGAAGGTCTTGCAGGAG
IL-4Rα rev	CAAGAGGACCTCGAGCTAAGAGACCCTCATGTATGTG
IL-13Rα1 for	ACGGAAACTCAGCCACCTGTGACA
IL-13Rα1 rev	CTGAGAGGCTTTCTTCAGGTTTTC
IL-4Rα-TM rev	CCCACCATTCCTCGAGTCACTTGGTGATGCTG
IL-13Rα-TM rev	GGAGGGAATATAATAATCTCGAGTCATTTTAGG
Igκ - Halo - IL-13Rα1 for	CGGATCGAATTCCACCATGGAGACA
Igκ - Halo - IL-13Rα1 rev	CGTCGACCTGCGATATCTAGAGCTTTC
IL-4Rα-EC for	TTTGGATCCTTTAAGGTCTTGCAGGAGCCCACCTGCGTC
IL-4Rα-EC rev	TTTCTCGAGGTGCTGCTCGAAGGGCTCCCTGTAGGAGTT
IL-13Rα1-EC for	TTTTGGATCCACGGAAACTCAGCCACCTGTGACAAATTTGAGTGTC
IL-13Rα1-EC rev	TTTTCTCGAGTGTGGAATTGCGCTTCTTACCTATACTCATTTCTTGGCT
IL-4 mutants for	AAATTGGCAGGATCCCACAAGTGCGATATCACC
IL-4 KFR rev	GGTGCTCGAGTTATCAGCTCGAACACTTGCGAAATTTCTCTTTCATGATC
IL-4 RGA rev	GGTGCTCGAGTTATCAGGCGCCACACTTGAACCATTTCGACTGCATGATCACTCT TAGCC
IL-4 DN rev	GTGGTGCTCGAGTTATCAGCTCGAACACTTTGAGTCTTTCTCGTCCATGATC
IL-4 H59Y K84D for	AGCTGATCCGATTCCTGGACCGGCTCGACAGGAACCTC
IL-4 H59Y K84D rev	GAGGTTCCTGTCGAGCCGGTCCAGGAATCGGATCAGCTGC
IL-4 WT for	CTTTTGGCGGCGGCGGCGCATTCTGCCTTTGCGGGATCCCACAAGTGCGATATCA CCTTA
IL-4 WT rev	TCATTAATGGTGGTGATGGTGGTGATGATGCGCGGCCGCGCTCGAACACTTTGA ATATTT
IL-13 WT for	CTTTTGGCGGCGGCGGCGCATTCTGCCTTTGCGGGATCCCCAGGCCCTGTGCCTC CCTCT
IL-13 WT rev	TTCATTAATGGTGGTGATGGTGGTGATGATGCGCGGCCGCGTTGAACTGTCCCTC CCTAA

[a] for: forward primer, rev: reverse primer.

Supplementary Methods

Simulation conditions

At the beginning of a simulation run all particles are randomly distributed on the surface and, depending on the corresponding degree of labelling (DOL_1 and DOL_2), marked as visible or invisible by chance, followed by the execution of the main loop in which 6 steps are consecutively computed until the simulation time exceeds a predefined duration (t_{end}):

1. A possible dissociation of each existing ternary complex is probed by comparing a random value between 0 and 1 to the dissociation probability p_{d}.
2. For each particle of species 1 a new position is calculated on the basis of an independent displacement in x- and y-direction, respectively, that depends on equation 4 multiplied by a value that is randomly drawn from a normal distribution. If the new position is located outside the current compartment, a random value between 0 and 1 is compared to the hopping probability p_{hop} to check whether compartment switching is allowed. If hopping is forbidden, a new position is calculated as long as it is located in the current compartment. If a particle leaves the simulation space at a border, it re-enters immediately at the opposite side to mimic a spherical surface and to avoid boundary effects.

   $$\Delta x = \Delta y = \sqrt{2 \cdot D \cdot \Delta t_{\mathrm{sim}}} \qquad \textbf{Supplementary Equation 1}$$

   Here, D is the diffusion constant and Δx and Δy are the mean displacements in the corresponding directions of the particle. Δt_{sim} is the time-resolution of the simulation.

   Additionally, possible bleaching of each visible particle is calculated during this step by comparing a random value between 0 and 1 to the bleaching probability p_{bleach}. Each bleached particle gets marked as invisible and is not further considered for image generation.
3. A new position for each particle of species 2 is calculated in the same way, excluding particles that belong to a ternary complex. Those are assigned a new position in step 5.
4. For each particle of species 1 a list of species 2 particles located in the same compartment is generated. If the inter-particle distance of a particle-pair in the list is below or equal to the association threshold d_{a}, a random value between 0 and 1 is compared to the association probability p_{a} to check for ternary complex assembly.
5. Particles of species 2 that belong to a ternary complex are randomly placed around the position of the corresponding species 1 particle keeping a ternary complex inter-receptor distance d_{c}.
6. To emulate motion blurring of a single molecule experimental setup that arises from the finite acquisition time of an EMCCD camera, all positions of visible particles are saved until a virtual lag-time Δt_{lag} has expired. In this case an image of the current frame is generated by (i) merging all positions from all time-points since the frame has started, (ii) blurring with a 2-D Gaussian smoothing kernel (MATLAB function: “imgaussfilt”) to simulate diffraction at an objective aperture and (iii) adding Gaussian noise (MATLAB function: “imnoise”) to mimic different noise sources an EMCCD camera is subjected to.

Parameter estimation

The parameters used for simulations and the basis of their estimation are summarized in Supplementary Table 7.

Supplementary Table 7 Overview of simulation parameters

Parameter	Value [a]	Source
Δt_{sim}	8 µs	calculated
Δt_{lag}	32 ms	experimental setup
t_{end}	70 s	manually set
f_{img}	300 frames (9.6 s)	empirically determined
p_{a}	0.06	empirically determined
d_{a}, d_{c}	8 nm	estimated from structural data
p_{d}	$3.68 \cdot 10^{-5}$ (~RGA)<br>$1.84 \cdot 10^{-6}$ (WT)	estimated (RGA) / calculated (WT) from experimentally determined rate constants
p_{hop}	0.02 / 0.015	empirically determined (particles/complexes)
D	1.25 / 0.94 µm² s^{-1}	experimentally determined (particles/complexes)
p_{bleach}	$1 \cdot 10^{-7}$	experimentally determined
Compartment size	74.9 nm	from [3] and empirically determined
N_1	A: 0.5 molecules µm^{-2}<br>B: 0.65 molecules µm^{-2}	manually set<br>experimentally determined
N_2	A: 0.5 molecules µm^{-2}<br>B: 0.77 molecules µm^{-2}	manually set<br>experimentally determined
DOL_1	A: 100% / B: 42%	manually set / experimentally determined
DOL_2	A: 100% / B: 32%	manually set / experimentally determined

[a] A/B: Simulation runs that were evaluated to obtain inter-receptor distances and correlation lengths were performed with parameter-set B (experimentally determined), while for all others parameter-set A (manually set for simplicity reasons) was used.

The diffusion constant D (1.25 µm² s^{-1}) that represents the molecule's free diffusion constant in a barrier-free membrane was determined experimentally by single molecule tracking of diffusing transmembrane IL-4Rα in polymer supported membranes. The hopping probability p_{hop} (0.02) was obtained empirically by varying its value until step-length distributions (molecule displacements at 32 ms intervals = 1 frame) acquired from simulations matched experimentally derived distributions for IL-4Rα. Likewise an additional factor was estimated (0.75) to decrease the diffusion constant and the hopping probability for ternary complexes as their movement was experimentally observed to be slower.

The ternary complex dissociation probability p_{d} was calculated as previously derived[2]:

$$p_{\mathrm{d}} = 1 - e^{-k_{\mathrm{d}} \cdot \Delta t_{\mathrm{sim}}} \qquad \textbf{Supplementary Equation 2}$$

where k_{d} is the experimentally determined ternary complex dissociation rate constant and Δt_{sim} is the time-resolution of the simulation.

The probability p_{a} (0.06) of forming a ternary complex upon two molecules of different species getting closer than the association threshold was estimated to yield a fraction of about 50-60% of all species 1 particles being

part of a ternary complex when running the simulation with IL-4 wt k_{d}. The association threshold d_{a} (8 nm) was set simultaneously to fulfil the former criterion and for simplicity the same value was assigned to the ternary complex inter-receptor distance d_{c} (8 nm), which is consistent with structural data of the receptor chains.

The average 1-dimensional displacement of all particles determines the spatial resolution of the simulation and is, according to Supplementary Equation 1, dependent on the diffusion constant as well as on the time-resolution of the simulation. The latter (8 µs) was chosen as short as possible to achieve a spatial resolution (4.5 nm) significantly below the ternary complex association threshold on the one hand, and as large as feasible to minimize the needed computation time for a simulation run.

The corresponding parameters of the used MATLAB functions for image manipulation (blurring, noise addition) were optimized to finally generate images that resemble their experimentally obtained counterparts (i.e. single molecule evaluation leads to a comparable localization precision of simulated and experimentally derived images). Image creation was started only after a distinct number (f_{img}) of initial frames that was determined to be 300 (9.6 s) to ensure an established dynamic equilibrium between binary and ternary complexes.

Supplementary References

1. Codling, E.A., Plank, M.J. & Benhamou, S. Random walk models in biology. *J R Soc Interface* **5**, 813-834 (2008).
2. Chotard-Ghodsnia, R., Drochon, A., Duperray, A., Leyrat, A. & Verdier, C. Static and dynamic interactions between circulating cells and the endothelium in cancer, in *Cancer Modelling and simulation*. (ed. L. Preziosi) 243-267 (CRC Press, UK; 2003).
3. Murase, K. *et al.* Ultrafine membrane compartments for molecular diffusion as revealed by single molecule techniques. *Biophys J* **86**, 4075-4093 (2004).
4. Junttila, I.S. *et al.* Redirecting cell-type specific cytokine responses with engineered interleukin-4 superkines. *Nat Chem Biol* **8**, 990-998 (2012).
5. Kraich, M. *et al.* A modular interface of IL-4 allows for scalable affinity without affecting specificity for the IL-4 receptor. *BMC Biol* **4**, 13 (2006).
6. Tony, H.P., Shen, B.J., Reusch, P. & Sebald, W. Design of human interleukin-4 antagonists inhibiting interleukin-4-dependent and interleukin-13-dependent responses in T-cells and B-cells with high efficiency. *Eur J Biochem* **225**, 659-665 (1994).
7. Moraga, I. *et al.* Instructive roles for cytokine-receptor binding parameters in determining signaling and functional potency. *Sci Signal* **8**, ra114 (2015).
8. Riedl, J. *et al.* Lifeact: a versatile marker to visualize F-actin. *Nat Methods* **5**, 605-607 (2008).
